# The relationship between longer leukocyte telomeres and dNCR in non-cardiac surgery patients: a retrospective analysis

**DOI:** 10.1186/s12871-023-02183-0

**Published:** 2023-08-22

**Authors:** Chen Liu, Ke Ding, Mannan Abdul, Qing-Chun Sun, Zhen-Feng Zhang, Meng-Meng Dong, Liu Han, Ming-Sheng Dai, Hui-Lian Guan, Yuan Han, He Liu, Xue-Fen Chen, Jun-Li Cao

**Affiliations:** 1 Jiangsu Province Key Laboratory of Anesthesiology, Xuzhou Medical University, NO. 209 Tongshan Road, Yunlong District, Xuzhou City, 221004 Jiangsu Province China; 2 Department of Anesthesiology, Zhongda Hospital Southeast University, No. 87 Dingjiaqiao Road, Gulou District, Nanjing City, 210009 Jiangsu Province China; 3 Department of Anesthesiology, Nanjing First Hospital, Nanjing Medical University, No. 68 Changle Road, Qinhuai, District, Nanjing, 210006 Jiangsu Province China; 4 Department of Anesthesiology, Eye & ENT Hospital of Fudan University, NO. 83 Fengyang Road, Xuhui District, Shanghai City, 20031 Shanghai China; 5 Department of Anesthesiology, The First Affiliated Hospital of Nanjing Medical University, NO. 300 Guangzhou Road, Gulou District, Nanjing CityJiangsu Province, 210015 China; 6 Department of Anesthesiology, The Affiliated Hospital of Xuzhou Medical University, NO. 99 Huaihai Road, Quanshan District, Xuzhou City, 221002 Jiangsu Province China; 7 Department of Anesthesiology, Affiliated Huzhou Hospital, Zhejiang University School of Medicine & Huzhou Central Hospital, Huzhou City, 313003 Zhejiang Province China; 8 Department of Epidemiology and Health Statistics, School of Public Health, Shanghai University of Traditional Chinese Medicine, Shanghai, 201203 China

**Keywords:** Delayed neurocognitive recovery, Telomere length, Aging

## Abstract

**Background:**

Cognitive decline following surgery is a common concern among elderly individuals. Leukocyte telomere length (LTL) can be assessed as a biological clock connected to an individual lifespan. However, the mechanisms causing this inference are still not fully understood. As a result of this, LTL has the potential to be useful as an aging-related biomarker for assessing delayed neurocognitive recovery (dNCR) and related diseases.

**Methods:**

For this study, 196 individuals over 60 who were scheduled due to major non-cardiac surgical operations attended neuropsychological testing before surgery, followed by additional testing one week later. The finding of dNCR was based on a measured Z-score ≤ -1.96 on two or more separate tests. The frequency of dNCR was presented as the primary outcome of the study. Secondly, we evaluated the association between dNCR and preoperative LTL.

**Results:**

Overall, 20.4% [40/196; 95% confidence interval (CI), 14.7–26.1%] of patients exhibited dNCR 1-week post-surgery. Longer LTL was identified as a predictor for the onset of early cognitive impairment resulting in postoperative cognitive decline [odds ratio (OR), 14.82; 95% CI, 4.01–54.84; *P* < 0.001], following adjustment of age (OR, 12.33; 95% CI, 3.29–46.24; *P* < 0.001). The dNCR incidence based on LTL values of these patients, the area under the receiver operating characteristic (ROC) curve was 0.79 (95% CI, 0.722–0.859; *P* < 0.001). At an optimal cut-off value of 0.959, LTL values offered respective specificity and sensitivity values of 64.7% and 87.5%.

**Conclusions:**

In summary, the current study revealed that the incidence of dNCR was strongly associated with prolonged LTL. Furthermore, this biomarker could help identify high-risk patients and offer insight into the pathophysiology of dNCR.

**Supplementary Information:**

The online version contains supplementary material available at 10.1186/s12871-023-02183-0.

## Introduction

Patients undergoing non-cardiac surgery often experience perioperative neurocognitive disorder (PND) [1, 2], which is in turn associated with a range of complications that can lower overall quality of life, prolong hospitalization, compromise an individual's work capacity and daily function, and increase the risk of mortality [3]. Cognitive decline occurring between discharge and 1-month post-discharge is classified as delayed neurocognitive recovery (dNCR) [4]. However, the causes of dNCR incidence remain poorly understood, and complex neuropsychiatric testing is required to diagnose this condition.

Both age and preexisting cognitive decline have been identified as significant risk factors associated with PND incidence [5, 6]. Telomeres, DNA structures located at the ends of human chromosomes, are often evaluated as biomarkers associated with aging at the cellular level [7]. This human telomeric DNA consists of repeating -(TTAGGG)- sequences. Telomeres prevent DNA damage recognition at the end of chromosomes, but in most somatic cells, they shorten 50–150 base pairs during each S phase of the cell cycle [8]. This biological condition, which affects most somatic cells in the human body, shortens telomeres below a certain length, activating the DNA damage response and cellular senescence. It is caused by the absence of a telomere-elongating process [9]. So telomere length (TL) measurements can offer insight into the degeneration of these sequences between birth and the time of measurement [10]. Maintaining telomeric integrity is central to post-mitotic neuronal responses to genomic and oxidative stressors [11]. Leukocyte TL (LTL) values identified through analyses of peripheral blood samples are the focus of increasing research efforts to identify epigenomic markers associated with Alzheimer's disease and other age-related neurodegenerative conditions. This is due to the convenience of collecting blood samples [12]. Surprisingly, evidence suggests long and short TL values may contribute to amnestic mild cognitive impairment (aMCI) pathogenesis. TL testing can identify individuals more likely to develop cognitive impairment [13]. These prior results highlight the potential value of exploring associations between TL and PND incidence [5, 13]. Consequently, the hypothesis that elderly non-cardiac surgical patients with longer LTL values have a higher incidence of cognitive impairment prompted the design of the present study. The primary goal of these analyses was to explore the relationship between LTL and dNCR onset in this patient population.

## Methods

### Study design

This retrospective analysis was performed at the Affiliated Hospital of Xuzhou Medical University (Xu Zhou, China) from December 2016 - July 2017. The clinical research ethics committee of the Affiliated Hospital of Xuzhou Medical University approved this study (Certification No. XYFY2018-KL035-01 approval date: 26/7/2018), which was registered at https://register.clinicaltrials.gov (No: NCT03703973; Principal investigator: J.L.C.; date of registration: 12/10/2018). This research was carried out following the Helsinki Declaration of 1964. This study was granted a waiver of informed consent because all follow-up information and blood samples were derived from our earlier investigation on the relationship between anosmia and postoperative cognitive impairment. All participants in our previous research submitted their consent after receiving all the pertinent details. The informed consent form also clarified that the individuals' blood samples and other information would be utilized to further investigate postoperative cognitive impairment. All patient data remained confidential, and study populations were coded to ensure anonymity. This manuscript was compiled in strict adherence to applicable STROBE guidelines. This study examined blood samples and cognitive test results from 196 participants.

### Subject enrollment

From December 2016 to July 2017, elderly patients ≥ 60 years of age, referred for major non-cardiac and non-neurological surgical procedures under general anesthesia with an anticipated 5-day hospital stay, were screened for study inclusion. Patients were excluded on meeting any of the the following criteria: (1) significantly impaired hearing, vision, or motor skills (hemiplegia, etc.); (2) abnormal personality characteristics or psychological illnesses with the potential to interfere with the completion of baseline neuropsychological testing; (3) any history of neurological disorders or diseases including depression, schizophrenia, Parkinson's disease, multiple sclerosis, or senile dementia; (4) any surgical procedures or severe trauma within the last 12 months; (5) alcoholism, drug dependence, or severe physical illnesses; (6) vital organ dysfunction; (7) preoperative Mini-Mental Status Exam (MMSE) scores classified as follows: < 17 points for illiterate individuals, < 20 points for individuals with a primary school education, or < 34 points for individuals with secondary education or higher [14, 15]; (8) a Geriatric Depression Scale (GDS) grade > 2; or (9) an unwillingness to complete the study procedures or to comply with protocols.

### Neuropsychological testing

Standard neuropsychological testing was administered to each patient twice, with the baseline test the day before surgery and the follow-up exam a week later. The testing was carried out in a serene atmosphere in general care wards. The appropriate neuropsychological tests were selected based on two International Studies of Postoperative Cognitive Dysfunction (ISPOCD 1 and 2) [1, 16, 17]. In total, testing included 9 tests with 11 subscales covering cognitive domains associated with memory, psychomotor speed and dexterity, physical motor speed, attentional capacity, and perceptual-spatial function (Table 1). Further details regarding this testing approach have been published previously [18].

**Table 1:** Cognitive domains and neuropsychological tests

Domain	Tests
**Memory (short-term, intermediate-term)**	The Short Story module of the Randt Memory (immediate, delayed recall)
**Psychomotor speed**	Trail Making Test (Part A)
	Digit Symbol subtest of the Wechsler Adult Intelligence Scale-Revised (Chinese edition)
**Manual dexterity**	Grooved Pegboard Test (dominant and non-dominant)
**Attention and concentration**	Digit Span (forward and backward) subtests of the Wechsler Adult Intelligence Scale-Revised (Chinese edition)
**Speed and flexibility of Verbal thought process**	The Verbal Fluency Test
**Motor domain**	Finger tapping
**Perceptual-spatial function**	Block subtest of the Wechsler Adult Intelligence Scale-Revised (Chinese edition)

Memory, physical motor speed, perceptual-spatial functioning, psychomotor speed and dexterity, and attentional ability were all assessed using a neuropsychological testing battery. It had nine tests and eleven subscales

The same criteria were used to recruit 30 subjects who did not undergo surgery or anesthesia for dNCR measures. The same study team conducted Identical neuropsychological testing on these volunteer controls at the same intervals.

### LTL measurements

Patient blood samples were collected one day before surgery and stored at -80℃ for subsequent analysis. These samples were not subject to any other testing before this study. LTL values were computed using Cawthon's formula [19]. Briefly, for each sample, LTL was calculated by assessing the ratio of the telomere repeat (T) copy number to that of a single-copy gene (S) through qPCR using the SYBR Green dye (T/S ratio). All samples were analyzed two times for statistical analyses by operators blinded to patient sample details.

### dNCR detection

The study definition of dNCR used for these analyses was based on the ISPOCD 1 definition from two prior reports [1, 16, 17], with dNCR incidence being calculated as follows: (ΔX_i_–$\overline{\mathrm{\Delta XC} }$)/SD.$\overline{\mathrm{\Delta XC} }$. These changes (ΔX_i_) were calculated by subtracting preoperative scores from postoperative values for each task. The averaged difference value for controls ($\overline{\mathrm{\Delta XC} }$) was considered indicative of a systematic error and was computed similarly. Practice effects were then accounted for by subtracting ΔX_C_ from ΔX_i_, and the resulting score was divided by the standard deviation (± SD) for the change observed in the control group (SD.$\overline{\mathrm{\Delta XC} }$) as a means of controlling for expected variability. Individuals were believed to be suffering from dNCR when they exhibited a Z-score ≤ -1.96 on a minimum of 2 different neuropsychological tests out of 10 possible tests.

### Statistical analyses

Through the utilization of univariate analysis, outlier detection and distribution normality assessments were carried out. *T-tests* or Wilcoxon rank tests were applied to compare continuous variables (age, height, BMI, years of education, follow-up duration, MMSE scores, blood loss, anesthetic duration, surgery duration, total fluid administration, LTL). In contrast, Fisher's exact or chi-square tests were used to compare categorical variables (previous history of the disease, surgery type, and gender).

Relationships between LTL and dNCR incidence were examined using multivariate logistic regression analyses through a backward stepwise approach. Covariates in these models included established risk factors associated with dNCR, including age, sex, and level of education [1, 17]. A ROC curve was used to examine the relationship between LTL and dNCR incidence. Youden's index was used to determine the best cut-off value to categorize patients into groups with longer and shorter LTL values. In these groups, the incidence rates of dNCR were then compared. All statistical analysis was performed using SPSS v 22.0 (IBM, NY, USA).

While this was a prospective analysis, sample size calculations were performed as a component of the study design process using PASS (v 11.0;NCSS, USA) based on two-independent proportions with odds ratios (OR). Based on prior studies of PND and correlations between this condition and changes in gene expression and olfactory dysfunction, dNCR was predicted to affect 20% of patients, with a risk of cognitive decline for individuals with shorter LTL values (T/S ratio < median T/S ratio) being twofold higher (OR = 3.0) as compared to individuals with longer LTL values (T/S ratio > median T/S ratio) [13]. At a 0.05 significance level and 0.80 power, a minimum of 190 patients would thus need to be enrolled to provide this study with sufficient statistical power.

## Results

### Study cohort

In our earlier investigation, 212 patients over 60 who were scheduled for major non-cardiac surgery underwent neuropsychological screening one day before surgery and one week postoperative. Of these patients, peripheral blood samples were unavailable for 16 individuals, while the remaining 196 were included in the present study. Table 2 compiled patient baseline demographic, surgical, and clinical details. Patients who developed dNCR were older than those who did not (*P* = 0.020), while no other demographic variables differed significantly between these groups.

**Table 2:** Clinical, surgical, and demographic characteristics of the dNCR and non-dNCR groups

	dNCR Group (*n* = 40)	non- dNCR Group (*n* = 156)	*P* value
Demographics			
Age, year	70.20 (7.74)	67.51 (5.74)	0.020^*^
Female sex, n (%)	14 (35.00)	52 (33.33)	0.843
Height, cm	164.75 (6.64)	165.29 (7.61)	0.563
Weight, mean (SD), kg	66.51 (9.54)	66.44 (10.54)	0.703
BMI, mean (SD), kg∙m^−2^	24.56 (3.55)	24.28 (3.34)	0.696
Education, year	7.73 (3.84)	8.30 (3.90)	0.395
Follow-up, day	7 (2)	7 (2)	0.220
MMSE, score	25.63 (2.91)	26.45 (2.87)	0.081
Hb, g/l	130.25 (15.64)	130.46 (17.49)	0.657
Previous history, n (%)			
Hypertension	17(42.50)	66 (42.31)	0.983
Diabetes mellitus	7 (17.50)	18 (11.54)	0.315
Coronary disease	2 (5.00)	23 (14.74)	0.100
Stroke	6 (15.00)	15 (9.62)	0.327
Smoking	6 (15.00)	33 (21.15)	0.386
Alcohol drinking	3 (10.00)	19 (12.18)	0.404
Type of surgery, n (%)			0.788
Urinary surgery	14 (35.00)	65 (41.67)	
Gastrointestinal Surgery	12 (30.00)	56 (35.90)	
Osteoarticular surgery	13 (32.50)	34 (21.79)	
Lung surgery	1 (2.50)	1 (0.64)	
Total fluid administration, ml	2220.00 (839.70)	2154.01 (565.00)	0.648
Blood loss, ml	209.50 (389.06)	194.81 (186.26)	0.435
Duration of anesthesia, min	211.63 (69.85)	197.92 (71.86)	0.283
Duration of surgery, min	176.62 (67.38)	167.80 (67.55)	0.335
LTLs	1.1446 (0.1992)	0.9286 (0.2725)	< 0.001^**^

The mean (SD) is shown for the continuous variables. Categorical variables are displayed as frequency (%). Two independent sample T-tests for normally distributed and Mann–Whitney tests for non-normally distributed variables were used to compare continuous variables. Fisher's exact test, Pearson chi-square, and continuity correction tests were used to compare proportions

*Abbreviations*: *dNCR*  delayed neurocognitive recovery, *BMI* body mass index, *MMSE* Mini-Mental State Examination, *LTL* leukocyte telomere length

*P < 0.05. **P < 0.001

### Cognitive outcomes

Overall, 20.4% [40/196; 95% confidence interval (CI), 14.7–26.1%] of patients were diagnosed with dNCR 1-week after surgery using the selected battery of neuropsychological testing that was performed 7(2) and 7(2) days postoperatively in patients that did and did not exhibit dNCR, respectively (Table 2). Additional file 1: Supplementary Table 1 summarizes the results of baseline neuropsychological testing across all included tasks for patients in these two groups. Relative to individuals in the non-dNCR group, those in the dNCR group performed better in the Trail Making Test Parts (*P* = 0.025), the Short Story module of the Randt Memory (Delayed recall) (*P* = 0.020), Grooved Pegboard test (GPT) (dominant hand) (*P* < 0.001), and GPT (non-dominant hand) (*P* = 0.046) relative to patients in the non-dNCR group. Neuropsychological testing results across all included tasks for patients in these two groups at the one-week follow-up time point are provided in Additional file 2: Supplementary Table 2. Individuals affected by dNCR exhibited worse performance in the Short Story module of the Randt Memory (Immediate recall: *P* = 0.005; delayed recall: *P* = 0.003), Trail Making Test Parts A (*P* = 0.003), GPT (dominant and non-dominant hands, both *P* < 0.001), the Verbal Fluency test (*P* = 0.002), and the Block subtest (*P* = 0.015). Neuropsychological test scores for control study participants at both time points are compiled in Additional file 3: Supplementary Table 3.

### The association between LTL and dNCR

**Fig. 1:**
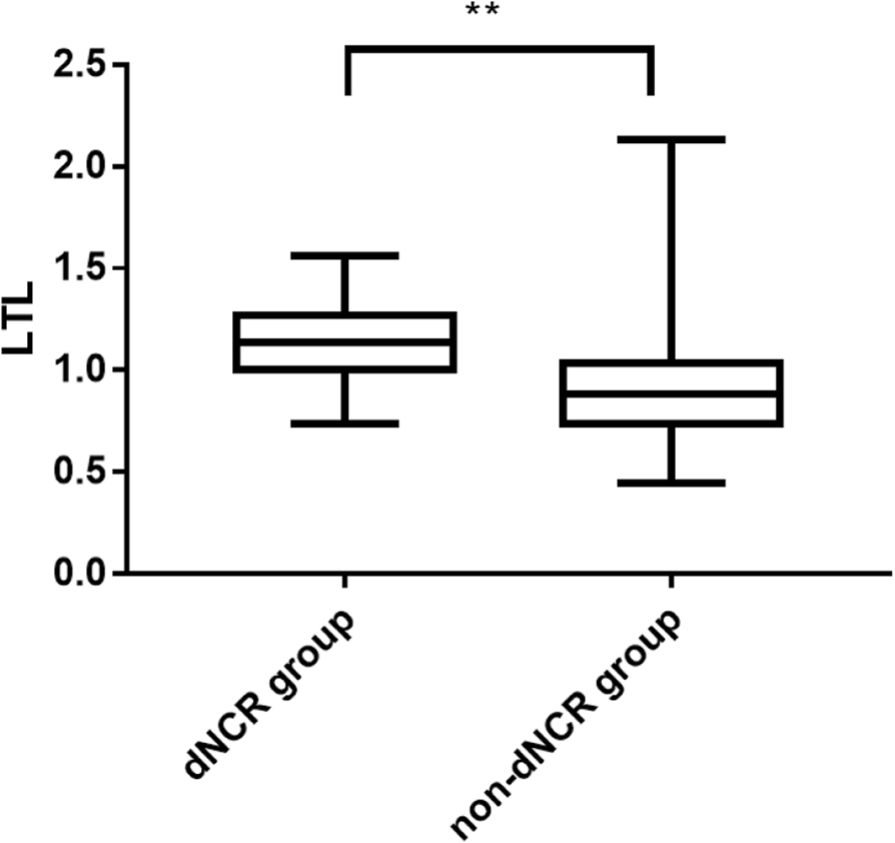
Comparison of peripheral blood LTL between the dNCR group and non- dNCR group. The median (interquartile range) LTL was markedly lengthier in patients who developed dNCR than in those who did not. **P < 0.001

**Fig. 2:**
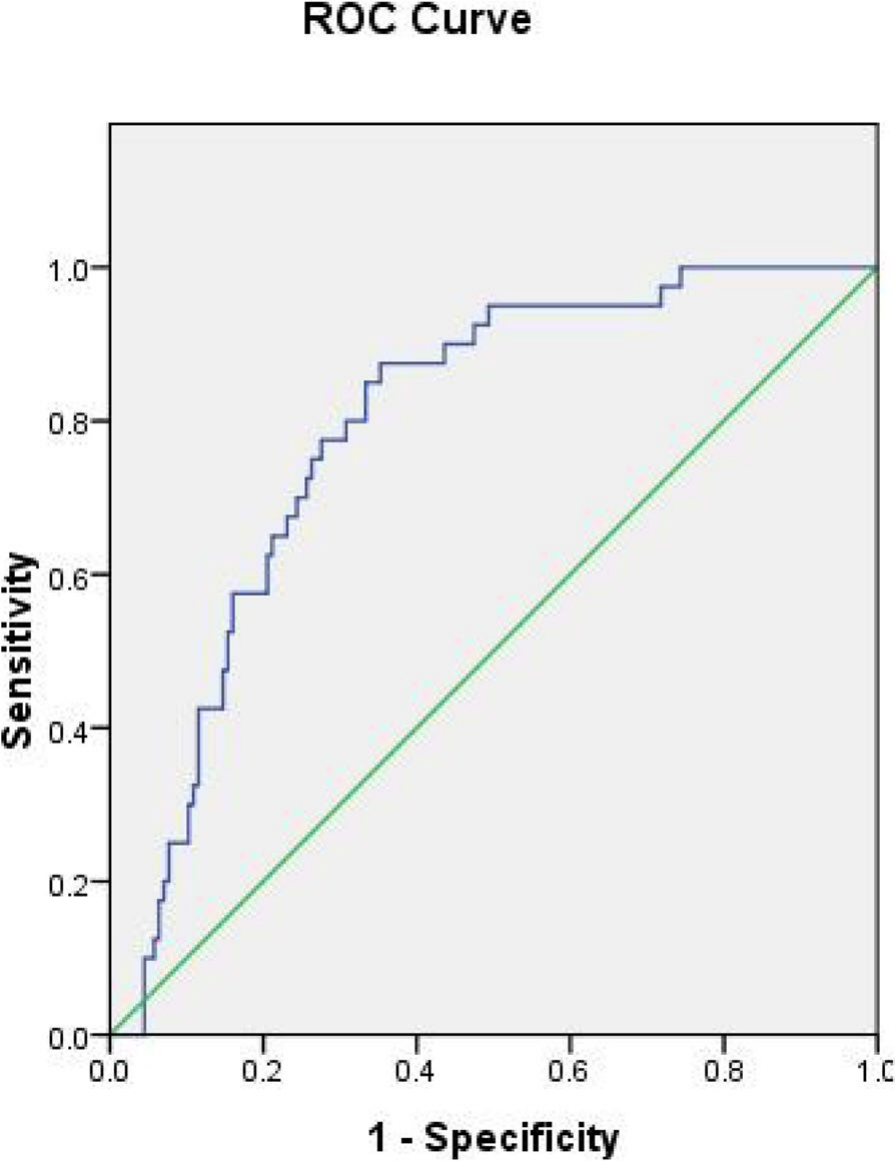
ROC curve for incidence prediction of dNCR in elderly patients following non-cardiac surgery. ROC curve analysis was performed for the LTL with dNCR. The area under the ROC curve of the duration of the LTL in the dNCR group was 0.791 (95% CI, 0.722–0.859). The optimal cut-off value was 0.959, with a sensitivity of 87.5% and a specificity of 64.7%

**Fig. 3:**
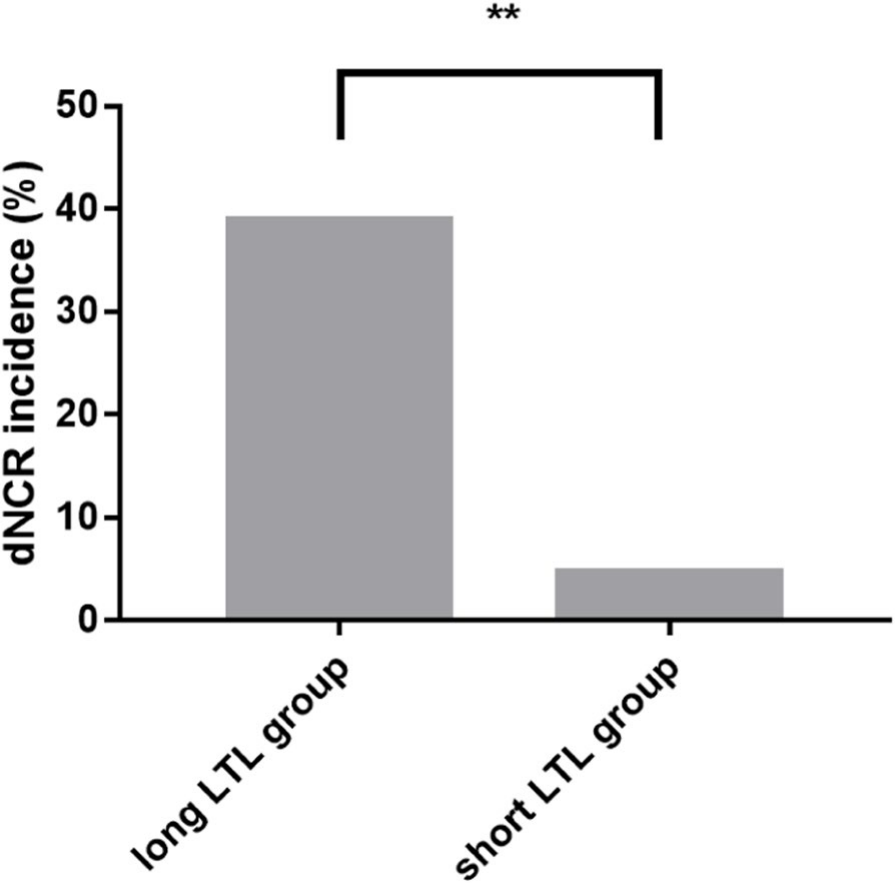
Comparison of the incidence of dNCR between long LTL group and short LTL group. All patients in the experimental group were categorized into the short LTL group and the long LTL group by the LTL median. The incidence of dNCR was compared between the two groups one week after the operation. The incidence of dNCR in the long LTL group was 7-fold higher than that in the short LTL group. **P < 0.001

Patients who developed dNCR experienced a considerably longer median LTL (interquartile range) than patients who did not (1.139 [1.005–1.272] vs. 0.880 [0.735–1.036]) (*P* < 0.001, Table 2, Fig. 1). In univariate analyses, longer preoperative LTL was identified as a predictor of dNCR detection one week postoperatively (OR, 14.82; 95% CI, 4.01–54.84; *P* < 0.001; Table 3), and this predictive strength remained even following adjustment for age (OR, 12.33; 95% CI, 3.29–46.24; *P* < 0.001; Table 3). When assessing the relationship between LTL and dNCR, the area under the ROC curve was 0.79 (95% CI, 0.722–0.859; *P* < 0.001). The optimal calculated cut-off value was 0.959, yielding respective sensitivity and specificity values of 87.5% and 64.7% (Fig. 2). Individuals in the long-LTL group exhibited a 7-fold higher rate of dNCR relative to individuals in the short-LTL group (Fig. 3).

**Table 3:** Associations between LTLs and dNCR

Predictors	Univariable Analysis^a^		Multivariable logistic regression analysis^b^	
	Odds Ratio (95% confidence interval)	*P* value	Odds Ratio (95% confidence interval)	*P* value
**LTL**	14.82(4.01–54.84)	< 0.001**	12.33(3.29–46.24)	< 0.001**
**Age**	1.07(1.01–1.13)	0.017*	1.05(0.99–1.12)	0.0696
**Sex**	0.93(0.45–1.93)	0.842	-	-
**Education**	0.96(0.88–1.05)	0.402	-	-
**MMSE**	0.91(0.81–1.02)	0.110	-	-

The relationship between LTL and the occurrence of dNCR was determined with multivariable logistic regression analysis. We included the LTL, significant perioperative variables (*P* ≤ 0.1), and the well-recognized dNCR risk factors, such as sex, age, and education levels, in existing studies in the multivariable logistic regression model. The backward logistic regression method was used

*Abbreviations*: *dNCR* delayed neurocognitive recovery, *LTL* leukocyte telomere length, *MMSE* Mini-Mental State Examination

^a^Occurrence of postoperative cognitive dysfunction was modeled as a function of a single predictor

^b^Occurrence of postoperative cognitive dysfunction was modeled as a function of all predictors

^*^P < 0.05; **P < 0.001

## Discussion

The results contradicted our initial predictions, showing that longer telomeres were associated with higher rates of dNCR after surgery under general anesthesia instead of telomere shortening, likely contributing to more severe postoperative cognitive impairment. This study is among the first to examine the relationships between TL and the early stages of PND, proving a foundation for future efforts to study the mechanistic basis for this neurodegenerative condition while offering a less invasive patient screening strategy.

According to previous findings, the average age of patients who developed dNCR and those who did not was significantly different in the current study (*P* = 0.020). Specifically, the average age of the dNCR group was 69.5 (66.00–77.00) years, while that of the non-dNCR group was 67 (62.00–71.00) years. This study recruited patients older than 60 years of age rather than 65 because the average life expectancy in China is shorter than that in Western countries.

Univariate analyses revealed longer preoperative LTL values as predictive of dNCR incidence 1 week after surgery (OR, 14.82, Table 3). These results remained significant after adjustment for patient age, education level, sex, and MMSE scores. In a multivariate logistic regression analysis, the OR for LTL was 12.33, so a ~12-fold increase in dNCR risk will be observed for every 1 unit increase in LTL. LTL has been proposed to provide value as a TL indication in different body sites [20]. Telomeres, which shorten with each cell cycle, serve as a mitotic clock that can be used to measure cellular proliferation and the number of cell divisions [21]. As such, LTL values may reflect a decrease in tissue volume in different brain regions [22, 23]; longer telomeres may mean less cell mitosis and larger brain size.

Several reports have similarly observed poorer cognitive performance in individuals exhibiting longer LTL values on functional language fluency and case memory tests [24]. In one analysis of 137 individuals affected by aMCI (mean age: 81.1 years; range: 70.9–90.8; 49.6% male), longer TL was associated with a higher level of aMCI [13], and some studies have reported that MCI patients exhibit a smaller hippocampal volume [25]. Thomas et al. found that relative to brains from control individuals, those from Alzheimer's patients showed significantly longer telomeres within the hippocampus and a concomitant reduction in hippocampal volume [22], suggesting that TL may represent an Alzheimer's disease-related biomarker [26].

The hippocampus serves as a central mediator of memory and learning-related activity. Hippocampal volume and function can be impacted by slower hippocampal cell proliferation in individuals exhibiting longer TL [27]. This model is potentially consistent with our present results, with the individuals with a longer LTL being more prone to dNCR and neurodegeneration due to less robust hippocampal cell proliferation.

Although contrary to conventional wisdom, long telomeres may be associated with aging. The latest published research paper in the top journal New England Journal of Medicine, on May 4, 2023, was published by researchers from the Hopkins University School of Medicine. The study also challenges universal cognition by demonstrating that too-long telomeres do not protect against aging and may even increase the risk for benign and malignant tumors [28].

In contrast to our present results, it has been reported that longer telomeres are related to healthy cognitive aging-, neurodegenerative disease-, and MCI-related outcomes [29, 30]. As such, fully understanding these telomeres' roles in this context remains somewhat challenging. Oxidative stress and inflammation are thought to influence TL variability, and both of these processes can play more direct pathogenic roles in Alzheimer's disease and cognitive aging [25, 31]. These contrasting results may also be attributable to individual differences or clinical settings. Even in age-matched populations, pronounced TL variability can also be observed [32], further complicating these studies. Studies of telomeric decline among individuals, rather than absolute TL, may represent a more effective means of assessing telomeric function, although further studies of this possibility are warranted.

There are some limitations to this study. Firstly, factors including pain scores, respiratory infection status, the stability of postoperative vitals, ICU admission, or patient Instrumental Activities of Daily Living (IADL) performance were not considered [4]. In addition, telomere length measurements were made using peripheral blood samples rather than neurons, given the difficulties of collecting neural tissue samples. However, peripheral blood telomere length is generally accepted as an indicator associated with the length of telomeres in other tissues. Moreover, LTL rather than telomere loss rate was the focus of this study, and telomere length can vary markedly at the individual level, particularly among older adults [32]. Indeed, within-individual changes in telomere length are more likely to offer meaningful data than cross-sectional analyses assessing differences in telomere length within a given population [32, 33].

Furthermore, because of the controversy between TL and cognition, and the vast majority show short telomeres are associated with greater frequency or severity of neurologic disease, this study needs replication to confirm the findings. Lastly, this retrospective analysis only examined the associations between cognitive impairment and changes in LTL over a short postoperative period, contributing to the potential for selection bias. Future research efforts should thus focus on prospective analyses of cognitive impairment over a more extended postoperative time period to more fully understand how telomere length relates to such cognitive decline.

## Conclusions

In conclusion, the association between LTL and dNCR incidence following non-cardiac surgery in an aged population was explored in this retrospective investigation. These analyses revealed that LTL measurements made using peripheral blood samples offer value as a predictor of dNCR development. However, further research will require further research on the mechanisms behind this association between longer baseline LTL and postsurgical cognitive impairment.

### Supplementary Information

**Additional file 1: Supplementary Table 1.** Demographic and clinical characteristics of the control group and all patients.

**Additional file 2:** **Supplementary Table 2.** Baseline neuropsychological test results.

**Additional file 3: Supplementary Table 3.** Neuropsychological test results at 1 week follow-up.

**Additional file 4: Supplementary Table 4.** Neuropsychological test results of the control group (*N* = 30).

## Data Availability

The corresponding author can provide all the mentioned data in the study, excluding the patients' personal information, to maintain privacy and confidentiality.

## References

[1] Moller JT, Cluitmans P, Rasmussen LS (1998). Long-term postoperative cognitive dysfunction in the elderly ISPOCD1 study. ISPOCD investigators. International Study of Postoperative Cognitive Dysfunction. Lancet.

[2] Chen L, Dong R, Lu Y (2019). MicroRNA-146a protects against cognitive decline induced by surgical trauma by suppressing hippocampal neuroinflammation in mice. Brain Behav Immun.

[3] Steinmetz J, Christensen KB, Lund T (2009). Long-term consequences of postoperative cognitive dysfunction. Anesthesiology.

[4] Evered L, Silbert B, Knopman DS (2018). Recommendations for the nomenclature of cognitive change associated with anesthesia and surgery-2018. Br J Anaesth.

[5] Dokkedal U, Hansen TG, Rasmussen LS (2016). Cognitive F unctioning After Surgery in Middle-aged and Elderly Danish Twins. J Neurosurg Anes thesiol.

[6] Evered L, Silbert B, Scott DA (2016). Cerebrospinal Flui d Biomarker for Alzheimer Disease Predicts Post-operative Cognitive Dysfunction. Anesthesiology.

[7] Mensa E, Latini S, Ramini D (2019). The telomere worl d and aging: Analytical challenges and future perspectives. Ageing Res Rev.

[8] Griffith JD, Comeau L, Rosenfield S (1999). Mammalian telomeres end in a large duplex loop. Cell.

[9] d'Adda di Fagagna F, Reaper PM, Clay-Farrace L (2003). Nature.

[10] Calado RT, Dumitriu B (2013). Telomere dynamics in mice and humans. Semin Hematol.

[11] Zhang P, Dilley C, Mattson MP (2007). DNA damage responses in neural cells: Focus on the telomere. Neuroscience.

[12] Forero DA, Gonzalez-Giraldo Y, Lopez-Quintero C (2016). Meta-analysis of Telomere Length in Alzheimer's Disease. J Gerontol A Biol Sci Med Sci.

[13] Roberts RO, Boardman LA, Cha RH (2014). Short and long telomeres increase risk of amnestic mild cognitive impairment. Mech Ageing Dev.

[14] Kirschbaum C, Hellhammer DH (1994). Salivary cortisol in psychoneuroendocrine research: recent developments and applications. Psychoneuroendocrinology.

[15] Liu HC, Chou P, Lin KN (1994). Assessing cognitive abilities and dementia in a predominantly illiterate population of older individuals in Kinmen. Psychol Med.

[16] Rasmussen LS, O'Brien JT, Silverstein JH (2005). Is perioperative cortisol secretion related to postoperative cognitive dysfunction?. Acta Anaesthesiol Scand.

[17] Rasmussen LS, Larsen K, Houx P (2001). The assessment of postoperative cognitive function. Acta Anaesthesiol Scand.

[18] Han Y, Han L, Dong M (2019). Preoperative Salivary Cortisol AM/PM Ratio Predicts Early Post-operative Cognitive Dysfunction After Non-cardiac Surgery in Elderly Patients. Anesth Analg.

[19] Cawthon RM (2002). Telomere measurement by quantitative PCR. Nucleic Acids Res.

[20] Takubo K, Izumiyama-Shimomura N, Honma N (2002). Telomere lengths are characte ristic in each human individual. Exp Gerontol.

[21] Leteurtre F, Li X, Gluckman E (1997). Telomerase activity during the cell cycle and in gamma- irradiated hematopoietic cells. Leukemia.

[22] Thomas P, O’Callaghan NJ, Fenech M (2008). Telomere length in white blood cells, buccal cells and brain tissue and its variation with ageing and Alzheimer's disease. Mech Ageing Dev.

[23] Wikgren M, Karlsson T, Lind J (2012). Longer leukocyte telomere length is associated with smaller hippocampal volume among non-demented APOE epsilon3/epsilon3 subjects. PLoS One.

[24] Harris SE, Deary IJ, MacIntyre A (2006). The association between telomere length, physical health, cognitive ageing, and mortality in non-demented older people. Neurosci Lett.

[25] Jones RW (2001). Inflammation and Alzheimer's disease. Lancet.

[26] Beauchet O, Launay CP, Sekhon H (2019). Association of hippocampal volume with gait variability in pre-dementia and dementia stages of Alzheimer disease: Results from a cross-sectional study. Exp Gerontol.

[27] Porter JB, Hoyes KP, Abeysinghe RD (1991). Comparison of the subacute toxicity and efficacy of 3- hydroxypyridin-4-one iron chelators in overloaded and nonoverloaded mice. Blood.

[28] DeBoy EA, Tassia MG, Schratz KE, et al. Familial clonal hematopoiesis in a long telomere syndrome. N Engl J Med. 2023;388(26):2422–33. 10.1056/NEJMoa2300503. 10.1056/NEJMoa2300503 PMC10501156 37140166

[29] Hagg S, Zhan Y, Karlsson R (2017). Short telomere length is associated with impaired cognitive performance in European ancestry cohorts. Transl Psychiatry.

[30] Liu M, Huo YR, Wang J (2016). Telomere Shortening in Alzheimer's Disease Patients. Ann Clin Lab Sci.

[31] Barnham KJ, Masters CL, Bush AI (2004). Neurodegenerative diseases and oxidative stress. Nat Rev Drug Discov.

[32] Mather KA, Jorm AF, Parslow RA (2011). Is telomere length a biomarker of aging? A review. J Gerontol A Biol Sci Med Sci.

[33] Cohen-Manheim I, Doniger GM, Sinnreich R (2016). Increased attrition of leukocyte telomere length in young adults is associated with poorer cognitive function in midlife. Eur J Epidemiol.

