# Supplementary material for: The relationship between longer leukocyte telomeres and dNCR in non-cardiac surgery patients: a retrospective analysis
Source: BMC Anesthesiol. 2023 Aug 22;23:284. doi: 10.1186/s12871-023-02183-0 (PMC10463441; doi:10.1186/s12871-023-02183-0)
Supplement: Supplementary file 1 — Additional file 1: Supplementary Table 1. Demographic and clinical characteristics of the control group and all patients. [file 12871_2023_2183_MOESM1_ESM.docx]

Supplementary Table 1. Demographic and clinical characteristics of the control group and all patients

|  | Control subjects (n=30) | All patients (n=196) | *P* value |
| --- | --- | --- | --- |
| **Age, year** | 69.50(64.00-76.00) | 67.00(63.00-72.00) | 0.184 |
| **Female sex（%）** | 12(40.00) | 66(33.67) | 0.497 |
| **Height, cm** | 167.50(162.75-173.00) | 165.00(160.00-170.00) | 0.098 |
| **Weight, mean (SD), kg** | 66.70(9.03) | 66.45(10.32) | 0.725 |
| **BMI, mean (SD), kg m^-2^** | 23.73(2.56) | 24.34(3.38) | 0.052 |
| **Education, year** | 9.00(9.00-12.00) | 9.00(6.00-9.00) | 0.079 |
| **Follow-up, day** | 7.00(6.00-7.00) | 7.00(6.00-8.00) | 0.009^*^ |
| **MMSE, score** | 27.50(25.75-29.00) | 27.00(24.00-28.00) | 0.296 |

The continuous variables are presented as mean (SD) if normally distributed or median (interquartile range) if non-normally distributed. Categorical variables are presented as frequency (%). Continuous variables were compared by two independent sample T-tests if normally distributed or Mann-Whitney tests for non-normally distributed variables. Proportions were compared by Pearson chi-square, continuity correction tests, or, Fisher's exact test. *Abbreviations*: *BMI* body mass index; *MMSE* Mini-Mental State Examination. **P* < 0.05.
