# Supplementary material for: The relationship between longer leukocyte telomeres and dNCR in non-cardiac surgery patients: a retrospective analysis
Source: BMC Anesthesiol. 2023 Aug 22;23:284. doi: 10.1186/s12871-023-02183-0 (PMC10463441; doi:10.1186/s12871-023-02183-0)
Supplement: Supplementary file 2 — Additional file 2: Supplementary Table 2. Baseline neuropsychological test results. [file 12871_2023_2183_MOESM2_ESM.docx]

Supplementary Table 2. Baseline Neuropsychological Test Results

| Test | Main Variables | dNCR patients (n=40) | Non- dNCR patients(n=156) | *P* value |
| --- | --- | --- | --- | --- |
| **The Short Story module of the Randt Memory** | Immediate recall score | 7.00(5.25-10.00) | 8.00(6.00-10.00) | 0.664^a^ |
|  | Delayed recall score | 8.00(4.25-10.00) | 9.00(7.00-11.00) | 0.020^a*^ |
| **Trail Making Test Parts A^#^** | Time, s | 63.00(47.50-87.25) | 53.00(38.00-69.00) | 0.025^a*^ |
| **Grooved Pegboard^#^** | Time, dominant hand, s | 113.00(90.25-146.50) | 89.50(72.25-107.00) | <0.001^a*^ |
|  | Time, nondominant hand, s | 108.50(83.25-156.50) | 95.00(82.00-120.00) | 0.046^a*^ |
| **Digit–Symbol subtest,mean (SD)** | Total score | 17.05(8.44) | 20.42(10.12) | 0.114^b^ |
| **Digit Span (forward and backward) subtests** | Total score | 10.00(8.00-11.00) | 10.00(8.00-11.00) | 0.517^a^ |
| **The Verbal Fluency test** | Total score | 33.00(24.25-39.00) | 35.00(28.00-45.75) | 0.179^a^ |
| **Finger tapping,mean (SD)** | Total score | 47.05(6.74) | 48.01(6.94) | 0.657^b^ |
| **Block subtest** | Total score | 6.00(3.00-7.00) | 6.00(2.00-7.00) | 0.461^a^ |

Data are presented as median (inter-quartile range) , unless otherwise indicated.

^#^ In timed tasks, lower scores reflect better performance.

**P* <0.05.

^a^ Mann-Whitney U test, ^b^ Independent sample T-tests.

*Abbreviations*: *dNCR* delayed neurocognitive recovery
