# Supplementary material for: The relationship between longer leukocyte telomeres and dNCR in non-cardiac surgery patients: a retrospective analysis
Source: BMC Anesthesiol. 2023 Aug 22;23:284. doi: 10.1186/s12871-023-02183-0 (PMC10463441; doi:10.1186/s12871-023-02183-0)
Supplement: Supplementary file 3 — Additional file 3: Supplementary Table 3. Neuropsychological test results at 1 week follow-up. [file 12871_2023_2183_MOESM3_ESM.docx]

|  | Main Variables | dNCR patients (n=40) | Non- dNCR patients (n=156) | *P* value |
| --- | --- | --- | --- | --- |
| **The Short Story module of the Randt Memory** | Immediate recall score | 9.00(6.00-10.00) | 10.00(8.00-12.00) | 0.005^*^ |
|  | Delayed recall score | 9.00(6.00-10.00) | 10.00(8.00-12.00) | 0.003^*^ |
| **Trail Making Test Parts A^#^** | Time, s | 67.00(50.25-90.75) | 52.50(41.00-74.00) | 0.003^*^ |
| **Grooved Pegboard^#^** | Time, dominant hand, s | 110.50(95.50-159.75) | 90.00(73.00-107.75) | <0.001^*^ |
|  | Time, nondominant hand, s | 122.50(102.75-172.50) | 98.00(80.25-121.00) | <0.001^*^ |
| **Digit–Symbol subtest, media(SD)** | Total score | 13.50(8.30) | 20.32(10.21) | 0.156 |
| **Digit Span (forward and backward) subtests** | Total score | 9.00(8.00-10.75) | 10.00(8.00-11.00) | 0.058 |
| **The Verbal Fluency test** | Total score | 27.50(21.00-41.75) | 35.00(28.00-46.75) | 0.002^*^ |
| **Finger tapping, media(SD)** | Total score | 43.70(8.08) | 48.19(7.42) | 0.956 |
| **Block subtest** | Total score | 5.00(2.00-6.00) | 6.00(3.00-8.00) | 0.015^*^ |

Supplementary Table 3. Neuropsychological Test Results at 1 Week Follow-up

Data are presented as median (inter-quartile range) , unless otherwise indicated.

Independent t tests or Mann-Whitney U test.

^#^ In timed tasks, lower scores reflect better performance.

^*^ *P* < 0.05.

*Abbreviations*: *dNCR* delayed neurocognitive recovery
