# Supplementary material for: The relationship between longer leukocyte telomeres and dNCR in non-cardiac surgery patients: a retrospective analysis
Source: BMC Anesthesiol. 2023 Aug 22;23:284. doi: 10.1186/s12871-023-02183-0 (PMC10463441; doi:10.1186/s12871-023-02183-0)
Supplement: Supplementary file 4 — Additional file 4: Supplementary Table 4. Neuropsychological test results of the control group (N = 30). [file 12871_2023_2183_MOESM4_ESM.docx]

Supplementary Table 4. Neuropsychological Test Results of the Control Group (N = 30)

| Test | Main Variables | Baseline | 1 week |
| --- | --- | --- | --- |
| **The Short Story module of the Randt Memory** | Immediate recall score | 7.50(5.00-11.00) | 11.00(8.00-13.00) |
|  | Delayed recall score | 11.00(6.75-13.00) | 12.00(9.00-13.00) |
| **Trail Making Test Parts A^#^** | Time, s | 64.00(53.25-98.00) | 67.50(51.50-105.00) |
| **Grooved Pegboard^#^** | Time, dominant hand, s | 121.00(90.75-150.50) | 103.50(89.25-138.75) |
|  | Time, nondominant hand, mean (SD), s | 128.17(39.94) | 118.90(36.11) |
| **Digit–Symbol subtest, mean (SD)** | Total score | 19.33(9.86) | 20.63(10.47) |
| **Digit Span (forward and backward) subtests, mean (SD)** | Total score | 10.13(2.11) | 10.20(2.04) |
| **The Verbal Fluency test, mean (SD)** | Total score | 36.87(12.55) | 40.47(14.98) |
| **Finger tapping, mean** | Total score | 46.00(42.75-52.25) | 48.50(41.00-52.00) |
| **Block subtest, mean (SD)** | Total score | 4.97(2.34) | 5.17(2.18) |

Data are presented as median (inter-quartile range) , unless otherwise indicated.

^#^ In timed tasks, lower scores reflect better performance.
